# Effect of storage temperature and equilibration time on polymethyl methacrylate (PMMA) bone cement polymerization in joint replacement surgery

**DOI:** 10.1186/s13018-015-0320-7

**Published:** 2015-11-17

**Authors:** Bryan T. H. Koh, J.H. Tan, Amit Kumarsing Ramruttun, Wilson Wang

**Affiliations:** Yong Loo Lin School of Medicine, National University of Singapore (NUS), 1E Kent Ridge Road, NUHS Tower Block Level 11, Singapore, 119228 Singapore; Department of Orthopedic Surgery, National University Hospital (NUH), 1E Kent Ridge Road, NUHS Tower Block Level 11, Singapore, 119228 Singapore

**Keywords:** Bone cement, PMMA, Temperature, Equilibration time, Handling characteristics, Setting time

## Abstract

**Background:**

In cemented joint arthroplasty, the handling characteristics (doughing, working, and setting times) of polymethyl methacrylate (PMMA) bone cement is important as it determines the amount of time surgeons have to optimally position an implant. Storage conditions (temperature and humidity) and the time given for PMMA cement to equilibrate to ambient operating theater (OT) temperatures are often unregulated and may lead to inconsistencies in its handling characteristics. This has not been previously studied. Hence, the purpose of this study was to investigate the effect of storage temperatures on the handling characteristics of PMMA cement and the duration of equilibration time needed at each storage temperature to produce consistent and reproducible doughing, setting, and working times.

**Methods:**

SmartSet® HV cement was stored at three different controlled temperatures: 20 °C (control), 24 °C, and 28 °C for at least 24 h prior to mixing. The cement components were then brought into a room kept at 20 °C and 50 % humidity. Samples were allowed to equilibrate to ambient conditions for 15, 30, 45, and 60 min. The cement components were mixed and the dough time, temperature-versus-time curve (Lutron TM-947SD, Lutron Electronics, Inc., Coopersburg, PA), and setting time were recorded. Analysis was performed using the two-way ANOVA test (IBM SPSS Statistics V.22).

**Results:**

At 20 °C (control) storage temperature, the mean setting time was 534 ± 17 s. At 24 °C storage temperature, the mean setting time was 414 ± 6 s (*p* < 0.001*) with 15 min of equilibration, 446 ± 11 s (*p* < 0.001*) with 30 min of equilibration, 501 ± 12 s (*p* < 0.001*) with 45 min of equilibration, and 528 ± 15 (*p* > 0.05) with 60 min of equilibration. At 28 °C storage temperature, the mean setting time was 381 ± 8 s (*p* < 0.001*) with 15 min of equilibration, 432 ± 30 s (*p* < 0.001*) with 30 min of equilibration, 487 ± 9 (*p* < 0.001*) with 45 min of equilibration, and 520 ± 16 s (*p* > 0.05) with 60 min of equilibration.

**Conclusions:**

This study reflects the extent to which storage temperatures and equilibration times can potentially affect the handling characteristics of PMMA cement. We recommend institutions to have a well-regulated temperature and humidity-controlled facility for storage of bone cements and a protocol to standardize the equilibration time of cements prior to use in the OT to improve consistency and reproducibility of the handling characteristics of PMMA cement.

## Introduction

Polymethyl methacrylate (PMMA) bone cement is an integral part of joint replacement surgery. It is used to secure the prosthetic implant components to a patient’s native bone. It stabilizes the prosthesis, fills the gap between the prosthesis and the surrounding bone, and transmits load from the prosthesis to the bone [1].

Many factors are known to influence the handling characteristics (doughing, setting, and working times) of PMMA cement [2–4]. These factors include mixing method, ambient temperature, and the composition of constituents in the cement [5–7]. The effect of temperature on the handling properties (doughing, setting, and working times) of PMMA cement has been well studied. Langdown et al. [7] showed that at ambient theater temperatures between 16 and 24 °C, the setting time could range between 10 and 20 min. However, variations within a given temperature range are large and can exceed 8 min. Pearson et al. [8] noted a 34 % reduction in the setting time with a rise in temperature from 20 to 25 °C. Farrar and Rose [3] reported that the time to reach a given viscosity is halved as the temperature increased from 19 to 25 °C. Other studies have also reported handling characteristics of PMMA cement at temperatures ranging from −14 to 6 to 37 °C [9].

Despite extensive published literature on the handling characteristics of PMMA cement, the relationship between storage temperature and equilibration time of the cement is unestablished. We believe that the storage conditions (temperature and humidity) of PMMA cement prior to use in the operating theater (OT) is often uncontrolled and unregulated, and the time given for the cement to equilibrate to ambient OT temperature is often overlooked by surgeons. This could potentially lead to inconsistencies in doughing, working, and setting times of the cement. Clinically, such inconsistencies may lead to PMMA cement setting before implants are positioned optimally, especially in complicated surgeries or when surgeons use instruments that they are unaccustomed to. On table revision, surgery may be required, which has implications on cost, duration of surgery, and morbidity of the patient [10]. Hence, the purpose of this study was to investigate the effect of storage temperatures on the handling characteristics of PMMA cement and the duration of equilibration needed at each storage temperature to produce consistent and reproducible doughing, setting, and working times, of which the latter had not been previously studied.

## Materials and methods

### Materials

The cement used was a commercially available high viscosity (HV) bone cement: SmartSet® HV (DePuy CMW, Blackpool, UK).

### Sample preparation

The powder and liquid components of the cement were kept in the manufacturer’s packaging in rooms with three different controlled temperatures: 20, 24, and 28 °C at 50 % humidity for at least 24 h prior to being mixed. All other tools and appliances used in mixing (spatula, mixing bowl, etc.) and temperature measurement (thermometer, thermocouple, PTFE-coated exotherm heat mold, etc.) were kept in controlled conditions that replicated the ambient OT environment in our institution at 20 °C and 50 % humidity. Just prior to mixing, the powder and liquid components of each cement pack was pre-portioned into two equal units according to the manufacturer’s recommended ratio of 20 g (powder):9.44 g (liquid) per unit. Graduated cylinders and an electronic analytical balance were used for pre-portioning the cement.

### Equilibration time

The pre-portioned powder and liquid components of the cement were brought into a room with temperature and humidity controlled at 20 °C and 50 %. The stopwatch was started, and the cement components were allowed to equilibrate at 15-min intervals (15, 30, 45, and 60 min), after which they were manually bowl mixed.

### Cement mixing

To establish a consistent time reference, timing began (*t =* 0 s) when the cement components were completely emptied from their respective packages into the mixing bowl. The powder component was first added to the mixing bowl followed by the liquid component. A spatula was then used to assist manual mixing of the components in a clockwise direction at a speed of 60 beats/min for 15 s until the powder was visually dissolved in the liquid.

### Doughing time

Dough time measurements were made in accordance with procedures detailed in ASTM F451-08. The mixture was probed at 15-s intervals with a non-powdered surgical-gloved (latex) finger 60 s after the onset of mixing. Dough time was recorded as the time when the cement separated cleanly from the gloved finger on probing.

### Setting time

**Fig. 1:**
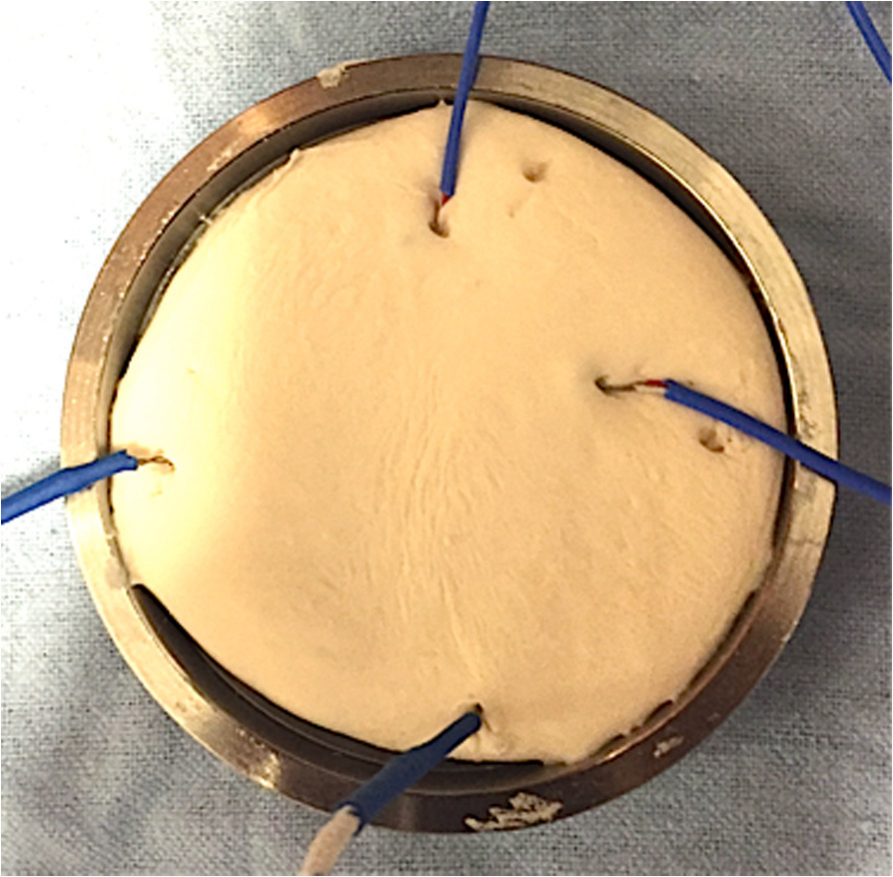
Temperature recording setup. Exotherm heat mold with a dimension of 7.5 cm (internal diameter) × 0.5 cm (depth) containing cement dough. Type K thermocouple wires were connected to Lutron TM-947SD (Lutron Electronics, Inc., Coopersburg, PA) for temperature-elapsed time recording. The tip of the K wires were inserted to a depth of 3.0 mm into the substance of the cement

Within 1 min after the dough time was reached, the cement dough was hand packed into a PTFE-coated exotherm mold in accordance with ASTM F451-08 dimensions (Fig. 1). Four type K thermocouple wires attached to a Lutron TM-947SD temperature data logger (Lutron Electronics, Inc., Coopersburg, PA) were placed at four corners in the substance of the cement at a depth of 3.0 mm. All temperatures were recorded at a rate of 0.5 Hz until cooling was observed. Prior to temperature recording, the thermocouple wires were calibrated in ice water (0 °C) and boiling water (100 °C) against a mercury-in-glass thermometer to an accuracy of ±1 °C.

**Fig. 2:**
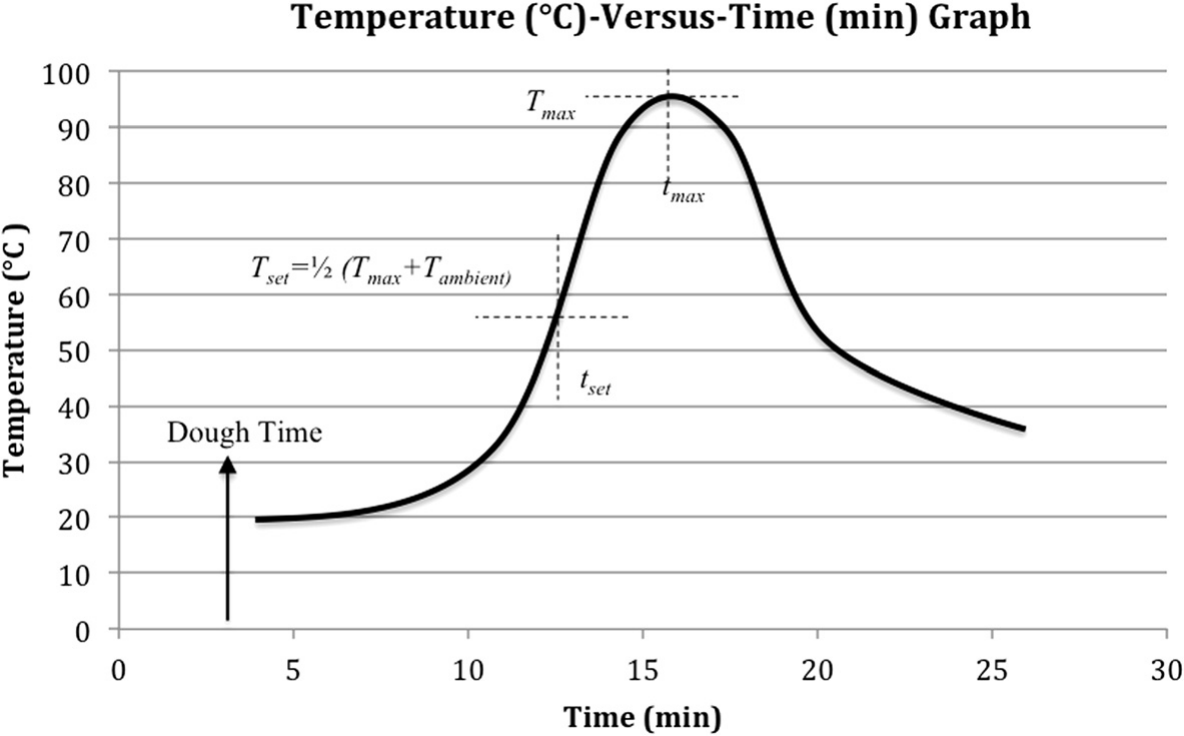
Theoretical temperature (°C) vs. time (min) curve of PMMA cement. This graph is a schematic representation of the shape of the cement temperature-versus-time curve that is consistent with ASTM F451-08. The temperature-versus-time curve was plotted and the setting time, *t*_set_ = setting time, *T*_set_ = setting temperature, *T*_max_ = maximum exothermic temperature, and *T*_ambient_ = ambient room temperature

The temperature-versus-time curve was plotted, and the setting time, *t*_set_, was determined from the curve by reading off the time at which the setting temperature, *T*_set_, as defined by the equation *T*_set_ = 1/2(*T*_max_ + *T*_ambient_) was reached in the ascending exotherm profile where maximum and ambient temperatures were *T*_max_ and *T*_ambient_, respectively (Fig. 2). Subsequently, subtracting the dough time from the setting time derived the working time of the cement.

### Data analysis

Test of significance of differences in the mean doughing, setting, and working times of the cement with respect to the equilibration time at the various storage temperatures was achieved using the two-way ANOVA test (IBM SPSS Statistics Version.22). Statistical significance was set at *p* < 0.05.

## Results

### Handling characteristics

**Fig. 3:**
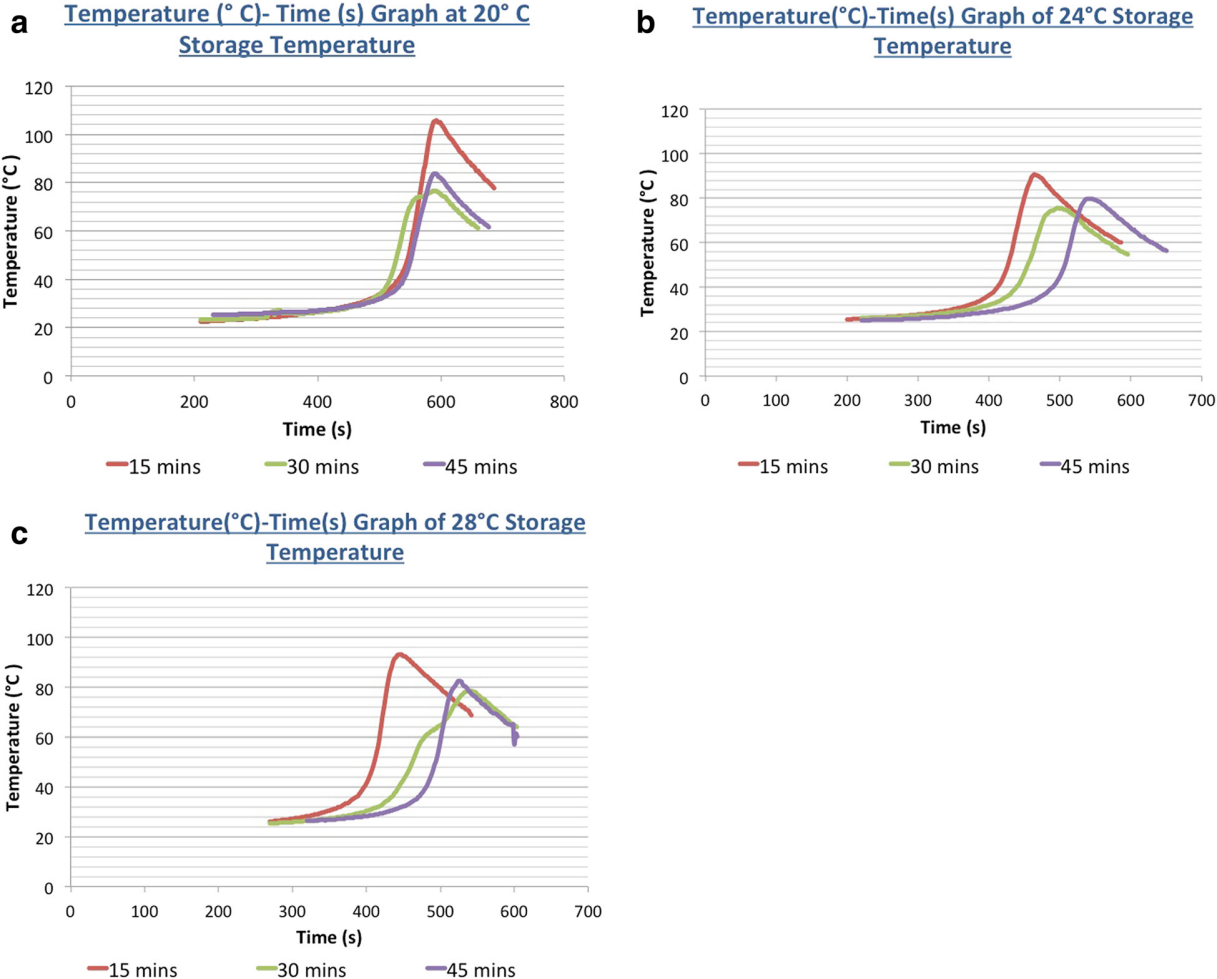
Temperature (°C)-versus-time (s) curves at different storage temperatures and equilibration times. **a** 20 °C storage temperature. **b** 24 °C storage temperature. **c** 28 °C storage temperature

The key finding of this study is that at the storage temperatures investigated (24 and 28 °C), the equilibration time significantly affects the handling characteristics (doughing, working, and setting times) of SmartSet® HV cement (Table 1). Figure 3a–c shows graphs of the temperature-versus-time relationship of the cement during polymerization using one representative sample at each storage temperature and equilibration time.

**Table 1:** Handling characteristics at different test conditions

	20 °C (control)			24 °C				28 °C			
Equilibration time (min)	15	30	45	15	30	45	60	15	30	45	60
Mean doughing time (s)	148 ± 7			97 ± 4	125 ± 6	152 ± 5	150 ± 4	90 ± 5	105 ± 8	157 ± 6	155 ± 6
Mean setting time (s)	534 ± 17			414 ± 6	446 ± 11	501 ± 12	528 ± 15	381 ± 8	432 ± 30	487 ± 9	520 ± 16
Mean working time (s)	386 ± 21			317 ± 6	321 ± 12	349 ± 13	374 ± 17	291 ± 9	326 ± 31	330 ± 13	355 ± 19

Table showing the handling characteristics (doughing, working, and setting times) of the bone cement stored at 24 and 28 °C at various equilibration times. Note that even though the mean setting time of the cement stored at 24 and 28 °C required 60 min of equilibration time to approximate the setting time of the control, only 45 min was required for the doughing time to equilibrate

In the control setup, the mean dough time achieved was 148 s (2.5 min) and the mean setting time achieved was 534 s (8.9 min).

**Fig. 4:**
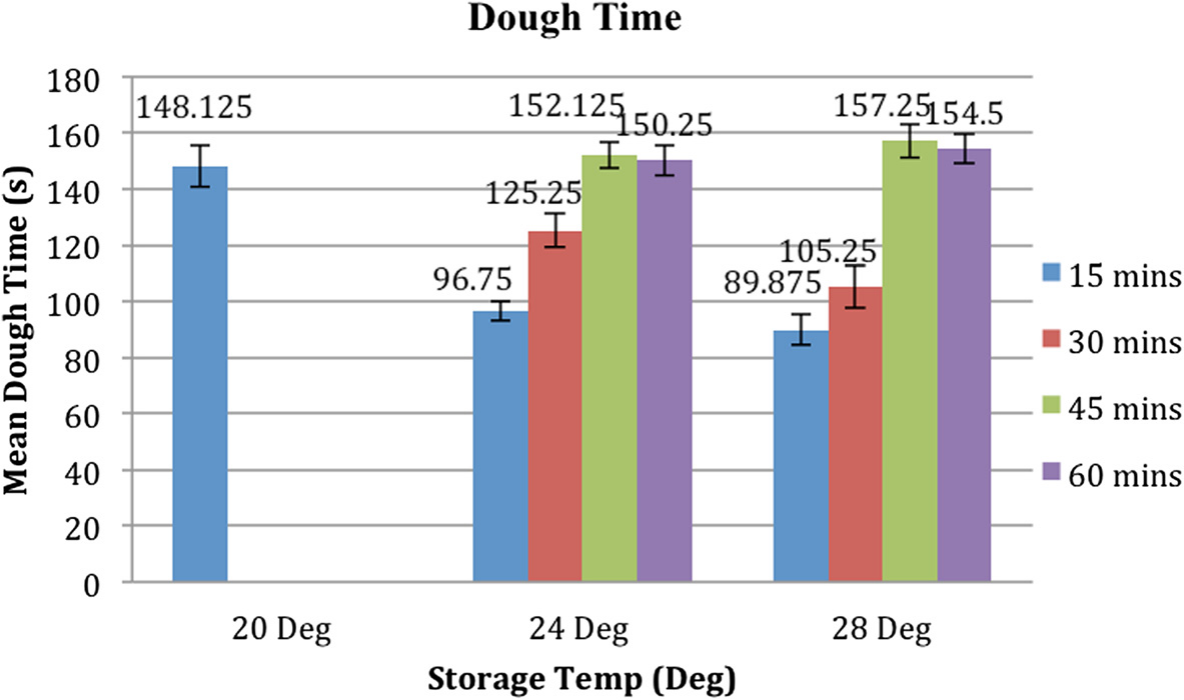
Dough time (s) at different storage temperatures and equilibration times. This graph shows the relationship between the mean doughing time (s) and storage temperature (°C) of the acrylic bone cement at various equilibration times. Note that at least 45 min was the required equilibration time of the cements stored at 24 and 28 °C for doughing time to be consistent with the control

At storage temperatures of 24 and 28 °C, the dough time was found to be consistent with dough times of the control setup after 45 min of equilibration at 20 °C. However, the difference in dough time could vary by up to 50 % between 15 and 45 min of equilibration (Fig. 4).

**Fig. 5:**
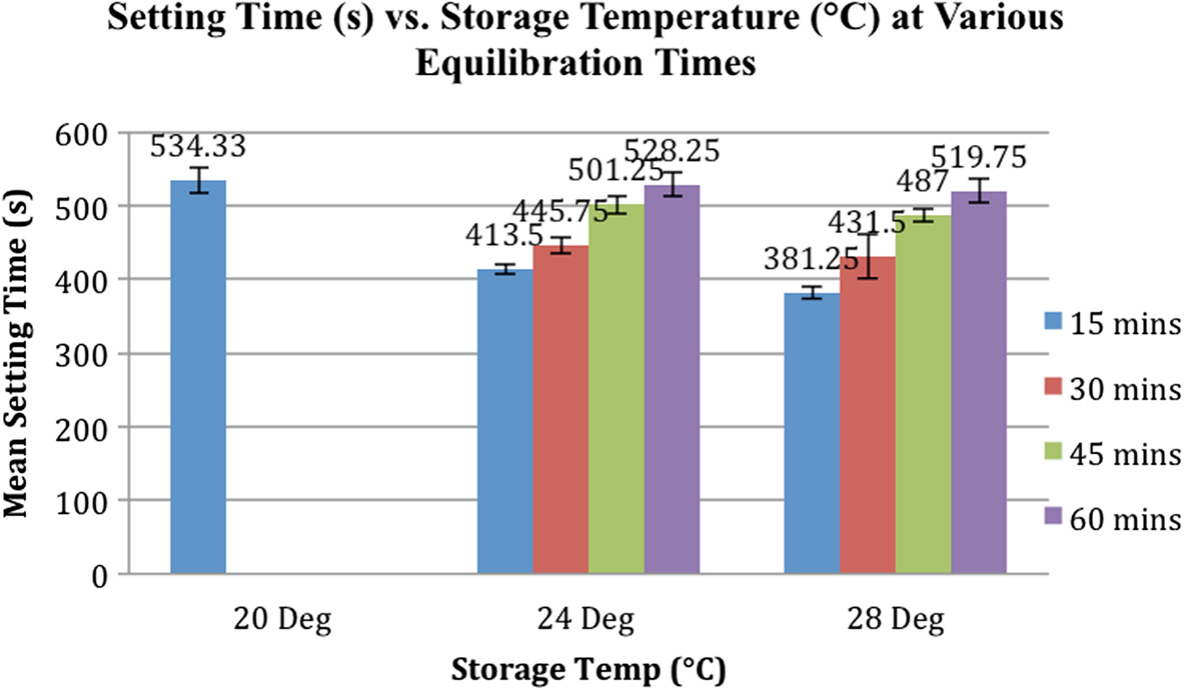
Setting time (s) at different storage temperatures and equilibration times. This graph shows the relationship between the mean setting time (s) and storage temperature (°C) of the acrylic bone cement at various equilibration times. Note that a minimum equilibration time of 60 min was required for the setting time of the cements stored at 24 and 28 °C to be statistically insignificant (*p* value >0.05) when compared to the control of 20 °C

At a storage temperature of 24 °C, cement samples that were allowed to equilibrate in a 20 °C room for 15 min had a mean setting time of 413 s (6.9 min; *p* value <0.001). When the equilibration time increased to 30 and 45 min, the mean setting time of the cement samples increased to 446 s (7.4 min; *p* value <0.001) and 501 s (8.4 min; *p* value <0.001), respectively. Only after an equilibration time of 60 min did the mean setting time of the cement increase to 528 s (8.8 min) and became statistically insignificant (*p* value >0.05) compared to the control (Fig. 5).

At a storage temperature of 28 °C, cement samples that were allowed to equilibrate in a 20 °C room for 15 min had a mean setting time of 381 s (6.35 min; *p* value <0.001). When the equilibration time increased to 30 and 45 min, the mean setting time of the cement samples increased to 432 s (7.2 min; *p* value <0.001) and 487 s (8.1 min; *p* value <0.001), respectively. Only after an equilibration time of 60 min did the mean setting time increase to 520 s (8.7 min) and become statistically insignificant (*p* value >0.05) compared to the control (Fig. 5).

The working time of the cement samples followed a similar temperature-equilibration time relationship at the different storage temperatures.

### Peak exothermic temperature

The maximum exothermic temperatures achieved in this study ranged from 57 to 108.2 °C. There was no apparent relationship between the storage temperature, equilibration time, and the maximum exothermic temperature achieved.

## Discussion

Many studies have investigated the relationship between ambient OT temperatures and the effect of pre-cooling cement at extremely low temperatures on the handling properties of bone cement [5–9]. Despite this, one entity that is often overlooked by surgeons in the clinical setting is the storage conditions of the bone cement prior to use in the OT and the time allowed for the cement to equilibrate to OT conditions. Knowledge on the storage conditions and equilibration time of the cements is of particular importance in tropical climates or in countries with calendar seasons where environmental temperatures can fluctuate by over 10 °C in months [11]. In such instances, storage of cement packages in unregulated and uncontrolled conditions may result in inconsistencies in estimating the setting and working times of the cement. This clinical problem is further exacerbated by variations in the time allowed for cement packages to equilibrate to ambient OT temperatures. This could potentially lead to greater inconsistencies in the handling characteristics of bone cement, which could prove detrimental for patients [7]. Hence, rather than relying on an abstract understanding of the handling properties of bone cement, the aim of our research was to empirically define the range of doughing, setting, and working times of bone cement at different storage temperatures and equilibration times.

Our results suggest that the duration of equilibration needed for bone cement to achieve consistent setting times at a controlled ambient OT temperature (20 °C) is directly proportional to its storage temperature. Clinically, this may translate into up to 26 % variation in the working time of bone cement, which is especially significant, considering that individual variation in the setting and working times of bone cements have been reported to exceed more than 50 % within a given temperature range [3, 7].

In addition, our results suggest that the doughing time of bone cement seems to be more sensitive to temperature changes than the setting time. We found that the difference in doughing times between 15 and 45 min of equilibration can vary up to 50 %. This is especially relevant in procedures that require syringe or cement gun extrusion of bone cement such as in hip arthroplasty and vertebroplasty [3, 5].

Although the findings of our study should not be extrapolated to the class of PMMA cements as a whole, as different cement preparations are known to have varying handling properties, the trend in our present results suggests strongly that storage temperature and equilibration times have a profound effect on the doughing and setting times of PMMA bone cement. Larger studies with a wider range of commercially available cements that investigate more handling properties such as viscoelastic parameters and a wider range of storage temperatures would be more conclusive.

## Conclusion

The present study results are promising in that they empirically defined the extent to which storage temperatures and equilibration times can potentially affect the handling characteristics of PMMA cement. The results also serve to re-emphasize the importance for surgeons to be aware of the storage conditions and duration of equilibration of bone cement when performing cemented orthopedic procedures.

We strongly recommend institutions to have a well-regulated and controlled storage facility for storage of bone cements and a strict protocol to standardize the equilibration time to improve consistency and reduce variations in the handling properties of PMMA cement.
